# On the interrelation between reduced lateralization, schizotypy, and creativity

**DOI:** 10.3389/fpsyg.2014.00813

**Published:** 2014-07-28

**Authors:** Annukka K. Lindell

**Affiliations:** School of Psychological Science, La Trobe UniversityMelbourne, VIC, Australia

**Keywords:** laterality, creativity, schizotypy, schizophrenia, hemisphere

Genius and madness have long been thought to be intimately entwined. However, the idea remains controversial: some rail against the stereotype of the mad scientist or the crazy artist (e.g., Schlesinger, 2009, 2012), while others note higher incidences of mental illness amongst creative geniuses, including prize-winning authors, visual artists, and poets (e.g., Andreasen, 1987; Kaufman, 2000-2001; Nettle, 2006). Consistent with early ideas of a shared genetic basis (e.g., Lombroso, 1891; Galton, 1892), a growing body of research highlights a positive correlation between mental illness and heightened creativity (e.g., Rothenberg, 2001). The relationship between creativity and schizotypy warrants close examination, as greater creativity is associated with higher levels of schizotypal traits (e.g., Folley and Park, 2005). Atypical brain lateralization may play the causal role, being evident in people who are highly creative *and* in people who have high levels of schizotypal traits (e.g., Weinstein and Graves, 2002). This paper argues in favor of the opinion that atypical lateralization prompts a cognitive processing style that enhances both creativity and schizotypy, suggesting a potential biological foundation for the link between genius and madness.

## Schizotypy and creativity

Conceptually, schizotypy represents the presence of schizophrenic-like thought patterns and/or belief systems in the absence of psychosis, including traits such as magical thinking, unusual perceptual experiences, and paranormal beliefs (American Psychiatric Association, 2013). Such traits index highly with a variety of unusual behaviors, prompting others to describe the schizotypal personality as “odd” or “eccentric” (Fisher et al., 2004). Although schizotypy is associated with vulnerability to schizophrenia (Lenzenweger, 2011), it is also linked with enhanced creativity: people involved in creative professions, such as musicians and visual artists, gain higher scores on measures of schizotypy than those in non-creative professions (Brod, 1997; Schuldberg, 2000–2001; Preti and Vellante, 2007; Gibson et al., 2009). Similarly, people with normal but high levels of schizotypal traits gain higher scores on a variety of measures of creativity, including conceptual expansion (drawing animals that reside on another planet), creative imagery (inventing and assembling an object from 3-dimensional figures; e.g., Abraham and Windmann, 2008), and the Torrance Tests of Creative Thinking (10 performance subtests that assess both verbal and non-verbal creative thinking; Poreh et al., 1994; see Thys et al., 2014, for review of the creativity assessment tools used to assess the relationship between creativity and psychopathology). Such findings index a close relationship between schizotypal traits, such as magical thinking and unusual perceptual experiences, and creative thinking.

## Schizotypy and cognitive style

Given that novelty forms a key component of creativity, an ability to think “outside the box” is a valuable characteristic of the creative mind. Whereas a wildly unconstrained, loosely-associated thinking style has a maladaptive manifestation in the disordered thinking symptomatic of schizophrenia, a moderate tendency toward linking remotely-associated concepts appears evolutionarily advantageous in that it promotes creative thinking. This may help explain the link between high levels of schizotypal traits and enhanced creativity: whereas disordered thinking in schizophrenia is beyond the individual's control, people with normal but high levels of schizotypal traits retain a greater degree of control over their cognitive processes (e.g., Lenzenweger, 2011). Thus, in schizotypy, the propensity to link remotely-associated concepts may serve to enhance creativity.

In assessing creativity, psychological research often relies on measures of divergent thinking, measuring “creativity” itself appearing too broad, too subjective, and perhaps simply ineffable. Divergent thinking is a flexible, open, and associative thinking style beneficial in solving complex problems and generating novel associations. Measures of divergent thinking confirm that people with normal but high levels of schizotypal traits show enhanced divergent thinking (e.g., Green and Williams, 1999), indicating superior ability in generating novel associations. This ability to draw connections between elements that initially appear to have nothing in common (Simonov, 1997) represents a fundamental component of creativity. In terms of semantic representation, it appears likely to result from “flatter” association hierarchies (i.e., more and broader associations to a stimulus); such hierarchies generate more creative solutions because they facilitate the drawing together of a wide range of information to solve a problem. In contrast, because “steeper” association hierarchies (i.e., fewer, more common associations to a stimulus; Grabner et al., 2007) are more focussed, activating a narrow range of the most common associations, they are less conducive to creative generation.

Research confirms that people with high levels of schizotypal traits activate flatter association hierarchies, allowing them to draw connections between distantly-related semantic associates. For example, people with higher magical ideation scores (a core component of measures of schizotypy) judge unrelated words to be more closely related than people with lower magical ideation scores (Mohr et al., 2001), suggesting facility in linking unrelated ideas. Gianotti et al. (2001) reported similar findings, indicating that people who believe in paranormal phenomena produce more original word associations than skeptics, suggesting looser semantic associations and a greater ability to link unrelated ideas (see also Pizzagalli et al., 2001). As heightened schizotypy is a predictor of increased paranormal belief (Hergovich et al., 2008), such findings appear highly consistent. The tendency to make links between unrelated or distantly-related concepts contrasts with the conceptual boundaries that typify “normal” thinking, but characterizes both schizotypal and creative thinking. Schizotypy and creativity are also linked by atypical cerebral lateralization, suggesting a potential causal link.

## Laterality, schizotypy, and creativity

The brains of people with schizophrenia evidence both structural and functional atypicalities, showing reduced hemispheric lateralization in comparison with healthy controls (see Lindell, 2011). Consistent with the proposed continuum between normal functioning and schizophrenia, with schizotypy representing “the less deviant bedfellow of ‘schizophrenia’,” (Claridge, 1997, p. 3), people with high levels of schizotypal traits also show evidence of reduced (e.g., Suzuki and Usher, 2009) or reversed (e.g., Rawlings and Claridge, 1984) hemispheric asymmetry. For example, Somers et al.'s (2009) meta-analysis of 10,058 participants found that higher levels of schizotypy were associated with increased incidence of non-right-handedness (particularly mixed handedness), indexing reduced lateralization. Dichotic listening data appear congruent, with people with high levels of schizotypal traits showing an atypical left ear (i.e., right hemisphere) advantage for dichotic listening (Poreh et al., 1994). Such findings imply greater than normal involvement of the right hemisphere in schizotypal individuals, consistent with research demonstrating a significant association between heightened creativity, schizotypy, and greater reliance on the right hemisphere (e.g., Weinstein and Graves, 2002). Atypical lateralization and greater involvement of the right hemisphere may help explain the heightened creativity associated with schizotypy.

The associational hierarchies described by Grabner et al. (2007) neatly match the semantic representational systems of the left and right hemispheres, being “steep” and “flat” respectively. Whereas activation in the left hemisphere spreads in a focussed manner, consistent with a more narrow, focussed semantic network and a steep associational hierarchy, activation in the right hemisphere spreads in a broader, more diffuse way, potentially facilitating links between distant associations in a flat associational hierarchy (e.g., Chiarello et al., 1990). Consequently, priming the left hemisphere with an ambiguous word (e.g., bank, scales) prompts activation of only the dominant meaning (e.g., money, weight); in contrast, priming the right hemisphere activates both dominant and subordinate associations (e.g., river, fish) (Burgess and Simpson, 1988).

Leonhard and Brugger (1998) argue that the right hemisphere's broad semantic representations play a causal role in creative and/or schizotypal thought processes; Folley and Park's (2005) functional imaging data are congruent. Folley and Park found that people with both high creativity (divergent thinking) and high levels of schizotypal traits preferentially recruited the right prefrontal cortex, contrasting with activation patterns observed in participants with schizophrenia and healthy controls. As the prefrontal cortex appears involved in processing novelty, such a finding appears entirely logical. Moreover, it implies that the ability to draw links between remotely associated concepts engages right hemisphere processes, consistent with the proposed diffuse semantic network in that hemisphere. Recent functional imaging data offer further support, highlighting a link between diffuse allocation of attention and heightened creativity (Takeuchi et al., 2011; Benedek et al., 2014). Overall, reduced hemispheric lateralization and diffuse attentional allocation appear beneficial for creativity, and indeed, are likely to play a causal role in the heightened creativity evident in people with high levels of schizotypal traits. A less lateralised brain may allow greater interhemispheric communication and transfer, facilitating the flat associational hierarchy that allows the drawing together of disparate concepts that promotes creative thinking (Lindell, 2011).

## Caveat

This opinion paper has argued that lateralization, schizotypy, and creativity are causally related, citing evidence of atypical hemispheric asymmetry in people with high levels of schizotypy and creativity (e.g., Weinstein and Graves, 2002; Folley and Park, 2005). However, it should be noted that findings in this literature are mixed, with some studies reporting no relationship between lateralization and schizotypy (e.g., Gooding and Braun, 2004). Inconsistencies in finding may be attributable to various factors, including differences in the measures used to assess schizotypy, the behavioral task(s) implemented, and the gender splits of the samples tested (see Schofield and Mohr, 2014, for discussion). Indeed, Schofield and Mohr's (2014) within-subjects investigation demonstrated that different schizotypy questionnaires produce inconsistent associations with behavioral measures of lateralization. Differences in finding may also arise from differences in the levels of schizotypal traits in the populations tested. Studies testing a schizotypy sample at the higher end of the normal spectrum may be more likely to report atypical lateralization than studies testing samples with schizotypy scores at the lower end of the range.

Methodological differences also contribute to inconsistencies in finding, as highlighted in Badzakova-Trajkov et al.'s (2011) study. Their functional imaging data indicated no correlation between magical ideation (a key component of measures of schizotypy) and hemispheric asymmetry; in marked contrast, their behavioral investigation found a negative correlation between magical ideation and handedness strength, indicating higher levels of schizotypal traits in mixed handers. This lack of consistency between functional imaging and behavioral findings highlights the need for further investigation, particularly as few studies have used imaging techniques to assess lateralization in people with normal but high schizotypy scores. Imaging investigations have instead focussed on clinical populations, assessing lateralization in people diagnosed with schizophrenia or schizotypal personality disorder. As investigations in the normal population have predominantly relied on behavioral techniques (e.g., visual half-field) and indirect measures of lateralization (e.g., handedness), there is a pressing need for structural and functional imaging investigation. Until such research is conducted, the proposed relationship between atypical lateralization, schizotypy, and creativity must be considered speculative.

## Conclusions

In the years since Leonhard and Brugger (1998) proposed that altered lateralization underlies both enhanced creativity and heightened schizotypy, the supporting data have grown. Schizotypy, creativity, and laterality appear intimately related, implying a common, presumably genetic, underlying mechanism. A cognitive bias toward broad processing, and drawing links between disparate concepts and apparently unrelated ideas, appears central to both the traits of schizotypy (e.g., magical ideation, perceptual abberations) and superior performance on measures of creativity (e.g., divergent thinking); this cognitive bias appears to reflect predominant right hemisphere processing.

At present there is a relative dearth of research assessing schizotypal traits, creativity, and right hemisphere activation/processing within the same population. Instead, the majority of the research has measured only two members of the triumvirate (e.g., schizotypy and creativity, or creativity and right hemisphere activation), allowing only speculative inferences about the interrelation of the three to be drawn. Simultaneous comparison of measures of all three components in the same population is needed to allow firmer conclusions.

Whilst the data imply a robust association between reduced lateralization, schizotypy, and creativity, the causal mechanism is presently unresolved. Genetic investigations presumably hold the key. For example, Mayseless et al. (2013) demonstrated a link between the dopaminergic system and creativity, with divergent thinking ability associated with polymorphism of the gene coding for DRD4 (dopamine receptor). Genome-wide investigations similarly show great promise, with Smalley et al. (2005) confirming genetic linkage between the regions coding for atypical cerebral asymmetry and disorders including autism, implying a shared phenotype. Similar genome-wide investigation is needed to investigate the presence of regions of linkage overlap in the genes for schizotypy, creativity, and atypical lateralization, potentially offering biological support for the proposed link between creative genius and madness.

### Conflict of interest statement

The author declares that the research was conducted in the absence of any commercial or financial relationships that could be construed as a potential conflict of interest.
